# Flavor quality evaluation system of Xinjiang milk knots by using SOM neural network and the fuzzy AHP

**DOI:** 10.1002/fsn3.1501

**Published:** 2020-03-21

**Authors:** Zhisheng Wei, Xueping Ma, Ping Zhan, Honglei Tian, Kaixuan Li

**Affiliations:** ^1^ College of Food Engineering and Nutritional Science Shaanxi Normal University Xi'an China; ^2^ Food College Shihezi University Shihezi China

**Keywords:** fuzzy analytic hierarchy process, sensory evaluation, SOM neural network, Xinjiang traditional milk knots

## Abstract

Self‐made milk knots in Xinjiang Kazakh ethnic group were used as material to establish the quality assessment system of flavor quality. The fuzzy analytic hierarchy process based on the optimal consistency matrix was used to evaluate the quality of the samples qualitatively and quantitatively. Its result is consistent with the cluster analysis of the SOM neural network. The results showed that the milk knot samples of Altay had differences with the milk knot samples of Yili. The comprehensive evaluation system is feasible and can evaluate the quality of milk knot samples by flavor characteristics. This can provide a reference for further research on the origin of differences between two types of milk knot samples.

## Graphical abstract

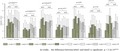

## 1 INTRODUCTION

Xinjiang milk knots are distinctive, high in nutrients, and always are considered as a good staple food (Azat et al., 2016). They are made by nomadic people with a natural fermentation process without any starter culture (Alegría, Szczesny, Mayo, Bardowski, & Kowalczyk, 2012). They are dry cheese with a sour or sweet taste. The sweet type is made of milk fat with much aroma while the sour type is less fatty and fragrant (Azat et al., 2016). The milk knots of Xinjiang have a high‐acid, sharp milky flavor and are thicker and darker than ordinary cheese (Alegría et al., 2012).

The overall scoring test was used in most of the sensory evaluation of milk knot in china, which makes the accuracy of the evaluation results extremely affected by the fact that the evaluation indicators were too simple. There were several studies on the sensory evaluation methods of milk knot in other countries, but there was no unified and complete system at present. Hailu et al. (2018) conducted training in four basic sensory methods of descriptive vocabulary (aroma, appearance, texture, and taste), selecting 10 well‐trained team members to do descriptive sensory analysis, the descriptive vocabulary of selection is silage, moldy, watery, firmness, sweet, salty, and sour. Ramírez‐López and Vélez‐Ruiz (2018) used the technique of “quantitative descriptive analysis” (QDA) to evaluate the texture of milk knot after processing 24 hr. The relationship between sensory and instrumental perception of the milk knot texture was determined by a 5‐point scale (from “zero” to “extreme”) which represents the perceived intensity of each texture attribute in conjunction with a description of the sensory analysis.

The fuzzy comprehensive evaluation result has vectority, which can reflect the fuzzy condition of the thing itself more accurately. It can be used for comprehensive evaluation of subjective indicators as well as objective indicators, especially in the comprehensive evaluation of subjective indicators. The evaluation effect of fuzzy comprehensive evaluation method was superior to other evaluation methods, so can be widely applied. Tanima and Madhusweta (2015) used fuzzy comprehensive evaluation to develop a mathematical model based on the fuzzy evaluation model (FCEM) and criticality analysis (FMECA) for evaluating the quality risk level in the food supply chain.

Artificial neural network (ANN), also known as the connector model, is a computing system developed on the basis of simulating human brain neural tissue. It is a kind of simulation of biological systems with the advantage of massive parallelism, distributed processing, self‐organization, and self‐learning, which widely used in pattern recognition, mapping relationship simulation, and so on.

In addition to parameter prediction, the use of ANN is primarily used to classify food types with some of their characteristics, and a high percentage of correlation was obtained in most studies. A study conducted by Correa, Montero Castillo, and Martelo (2018) and Song, Perez‐Cueto, and Bredie (2018) evaluated the characteristics of the beer brand, which was returned the class is “good” or “bad” through the ANN. The implementation of the ANN allowed the quality of the sample to be distinguished at 100% efficiency in the prediction of the beverage under study. On the other hand, Farzaneh's studies show that the ANN method not only has many applications in food research to solve different directions’ problems, but also has the characteristics of fast convergence, good effect, and low cost (Dolatabadi et al., 2016; Farzaneh, Bakhshabadi, et al., 2017; Farzaneh & Carvalho, 2017; Farzaneh, Ghodsvali, et al., 2017; Jabrayili et al., 2016; Moghimi, Farzaneh, & Bakhshabadi, 2018; Rostami, Farzaneh, Boujmehrani, Mohammadi, & Hamid, 2014).

This study aimed to (a) establish the quality assessment system of flavor quality in cheese samples qualitatively and quantitatively; (b) evaluate the flavor quality by the fuzzy analytic hierarchy process based on the optimal consistency matrix; and (c) indicate quality assessment system has certain feasibility by the SOM neural network clustering result.

## 2 MATERIALS AND METHODS

### 2.1 Materials

Nine batches of milk knot samples (two classes) were purchased from different local wholesalers in the main Kazakh settlements (Kazak Autonomous Prefecture of Ili and Altay Prefecture), where was in the northern Xinjiang Uygur Autonomous Region of China. The specific geographical location of these samples is shown in the Figure S1. All samples were labeled and transported at 4°C to the laboratory for analysis. Sensory analysis by the trained panelists was conducted within 1 month after the milk knots were purchased.

### 2.2 Sensory evaluation

Descriptive sensory testing was performed in Food College of Shihezi University. All milk knots were evaluated at normal temperature.

For flavor evaluation, a trained descriptive sensory panel (*n* = 20; 15 females and five males, ages 22–40 years) evaluated the milk knot in triplicate using a milk knot flavor lexicon (creamy aroma, sweet, oxidized aroma, whey, sour aroma, milky rancid, and waxy; Boris et al., 2018; Hailu et al., 2018; Hickey et al., 2018; Jo, Benoist, Ameerally, & Drake, 2018; Ramírez‐López & Vélez‐Ruiz, 2018). Panelists were trained and used the sensory intensity descriptors in Table S1 for experimental samples as described. All samples were stored at 4°C before being presented to the panel at normal temperatures and coded with a randomly selected three digit code. Evaluation was performed using the method that seven sensory attribute evaluation indicators were divided into 0–5 flavor intensity levels (Silva et al., 2018; Song et al., 2018). Each panelist had at least 150 hr of prior experience with descriptive analysis of flavor with various dairy products, including milk knot and yogurt. Milk knots were cut into 3 × 3 cm cubes, and 20 g were placed into 60‐ml cups. Four milk knots were evaluated in sessions, with an enforced 2‐min rest between samples. Replications were evaluated on different days (Yu, Low, & Zhou, 2018).

### 2.3 Data analysis

All multivariate analyses were performed on the MATLAB 2018b.

#### 2.3.1 Fuzzy analytic hierarchy process (AHP) based on optimal consistency matrix

Firstly, establishing fuzzy factor sets U and review sets V. The flavor quality factor set U, that is, the collection of sensory evaluation indicators of Xinjiang traditional milk knots' flavor. The review sets V, that is, product grade of sensory evaluation.

$$U=\left\{U_{1},U_{2},U_{3},U_{4},U_{5},U_{6},U_{7}\right\}\;\;V=\left\{V_{1},V_{2},V_{3},V_{4},V_{5},V_{6}\right\}$$

Among them.

$$U_{i}=\left\{\begin{array}{c} 1\to creamy\;\text{aroma}\\ 2\to \text{sweet}\\ 3\to oxidised\;\text{aroma}\\ 4\to whey\\ 5\to \text{sour aroma}\\ 6\to mil\text{k rancid}\\ 7\to waxy \end{array}\right.\;\;V_{i}=\left\{\begin{array}{c} 5\to \text{unbearable strong smell}\\ 4\to \text{strong}\\ 3\to \text{easy to feel the smell}\\ 2\to \text{very weak but can distinguish its nature}\\ 1\to \text{barely feel the smell}\\ 0\to \text{tasteless} \end{array}\right.$$

Then, the relative importance *W* of the flavor attribute evaluation index is obtained by the eigenanalysis method based on the optimal consistency matrix (shown as follows), which is the weight set.

This paper used the 5‐scale method to divide the evaluation criteria into five layers, namely −2, −1, 0, 1, and 2. Among these five levels, level 2 indicates that the former sample is better than the latter one, level 1 indicates that the former is better than the latter, 0 indicates that both are equally important, −1 indicates that the latter is generally better than the former, and −2 indicates that the latter is better than the former.

The pairwise comparison judgment matrix was established by the standard form $A={\left(a_{\mathrm{ij}}\right)}_{n\times n}$,

$$A=\left[\begin{array}{cccc} a_{11} & a_{12} & \cdots & a_{1n}\\ a_{21} & a_{22} & \cdots & a_{2n}\\ \vdots & \vdots & & \vdots \\ a_{n1} & a_{n2} & \cdots & a_{\mathrm{nn}} \end{array}\right].$$

Among them

$$a_{\mathrm{ij}}\in \left\{2,1,0,-1,-2\right\}$$

The matrix $T={\left(t_{\mathrm{ij}}\right)}_{n\times n}$ was the optimal transfer matrix of matrix *A*,

$$T=\left[\begin{array}{cccc} t_{11} & t_{12} & \cdots & t_{1n}\\ t_{21} & t_{22} & \cdots & t_{2n}\\ \vdots & \vdots & & \vdots \\ t_{n1} & t_{n2} & \cdots & t_{\mathrm{nn}} \end{array}\right].$$

Among them

$$t_{\mathrm{ij}}=\frac{1}{n}\sum_{k=1}^{n}\left(a_{\mathrm{ik}}-a_{\mathrm{jk}}\right)=\frac{1}{n}\sum_{l=1}^{n}\left(a_{\mathrm{il}}+a_{\mathrm{lj}}\right)$$

The matrix *T* was transformed into $F={\left(f_{\mathrm{ij}}\right)}_{n\times n}$ as the optimal consistency matrix, that is,

$$F=\left[\begin{array}{cccc} f_{11} & f_{12} & \cdots & f_{1n}\\ f_{21} & f_{22} & \cdots & f_{2n}\\ \vdots & \vdots & & \vdots \\ f_{n1} & f_{n2} & \cdots & f_{\mathrm{nn}} \end{array}\right].$$

Among them

$$f_{\mathrm{ij}}=exp\left(t_{\mathrm{ij}}\right)$$

Using the square root method to obtain the relative importance *W* of the corresponding index factors, $W=\left\{w_{1},w_{2},\cdots ,w_{n}\right\}$, that is,

$$W_{\mathrm{ij}}=\frac{\overset{-}{w_{i}}}{\sum_{j=1}^{n}\overset{-}{w_{j}}}$$

Among them

$${\bar{w}}_{l}\;=\;{\left(\prod_{k=1}^{n}f_{\mathrm{ij}}\right)}^{1/n}$$

Finally, the comprehensive evaluation results will be calculated by the formula *P = W·R*, where *W* is the weight set and *R* is the fuzzy matrix.

#### 2.3.2 SOM neural network for cluster analysis

Self‐organizing map (SOM) neural network is an unsupervised self‐organizing, self‐learning network. It can reduce the dimensionality of mapping from input space to output plane, and the mapping has topological feature retention properties (Chen & Kuo, 2019). It automatically finds the intrinsic characteristics of each input type by constantly adjusting the weights of network nodes, which is very effective for solving the problem that the input variables are not obvious, the feature parameters are interlaced and the nonlinear distribution (Yao, Tang, & Hu, 2018).

## 3 RESULTS AND DISCUSSION

### 3.1 Sensory analysis

The statistical results for the sensory evaluation of Xinjiang milk knots are summarized in Figure S2. The sensory values of the two labels significantly differed (*p* < .001) in total. This was our basic for further analysis. Only four attributes' intensity (creamy aroma, sweet, oxidized aroma, whey) had significant difference in seven attributes. The others (sour aroma, milk rancid, waxy) do not differ significantly. This finding may be attributed to the characteristics of Xinjiang Kazakh, and the most of Xinjiang milk knots have the characteristics of sour, milk rancid, and waxy flavor. It maybe results from *Pichia kudriavzevii*, which was the most predominant yeast species isolated from Kazak artisanal cheeses. And its generation of aroma compounds (ethanol, ethyl acetate, 3‐methyl butanol, and acetic acid) could lead to the formation of characteristic flavors in traditional cheese (Zheng et al., 2018).

### 3.2 Pairwise comparison judgment matrix

The pairwise comparison judgment matrix is shown in Table S2.

Relative importance (weight set) *W* is:

W=(0.4016,0.2268,0.1704,0.0723,0.0627,0.0354,0.0307).

The fuzzy matrix of each sample is obtained from the summary results:

$$R_{1}=\left[\begin{array}{cccccc} 0.00 & 0.10 & 0.20 & 0.35 & 0.30 & 0.05\\ 0.00 & 0.30 & 0.40 & 0.25 & 0.05 & 0.00\\ 0.00 & 0.30 & 0.30 & 0.20 & 0.20 & 0.00\\ 0.10 & 0.50 & 0.10 & 0.10 & 0.20 & 0.00\\ 0.05 & 0.15 & 0.30 & 0.35 & 0.15 & 0.00\\ 0.30 & 0.45 & 0.05 & 0.10 & 0.10 & 0.00\\ 0.30 & 0.35 & 0.30 & 0.00 & 0.05 & 0.00 \end{array}\right]\;\;R_{2}=\left[\begin{array}{cccccc} 0.15 & 0.40 & 0.25 & 0.00 & 0.15 & 0.05\\ 0.20 & 0.50 & 0.20 & 0.10 & 0.00 & 0.00\\ 0.20 & 0.50 & 0.15 & 0.10 & 0.05 & 0.00\\ 0.20 & 0.50 & 0.25 & 0.05 & 0.00 & 0.00\\ 0.30 & 0.40 & 0.10 & 0.10 & 0.10 & 0.00\\ 0.55 & 0.30 & 0.10 & 0.00 & 0.05 & 0.00\\ 0.35 & 0.30 & 0.25 & 0.10 & 0.00 & 0.00 \end{array}\right]R_{3}=\left[\begin{array}{cccccc} 0.05 & 0.05 & 0.25 & 0.40 & 0.15 & 0.10\\ 0.10 & 0.25 & 0.40 & 0.20 & 0.05 & 0.00\\ 0.10 & 0.10 & 0.40 & 0.30 & 0.10 & 0.00\\ 0.15 & 0.35 & 0.30 & 0.05 & 0.15 & 0.00\\ 0.10 & 0.15 & 0.45 & 0.15 & 0.15 & 0.00\\ 0.40 & 0.40 & 0.10 & 0.05 & 0.05 & 0.00\\ 0.15 & 0.35 & 0.35 & 0.15 & 0.00 & 0.00 \end{array}\right]\;\;R_{4}=\left[\begin{array}{cccccc} 0.05 & 0.10 & 0.30 & 0.25 & 0.20 & 0.10\\ 0.20 & 0.40 & 0.20 & 0.15 & 0.05 & 0\text{.0}0\\ 0.00 & 0.20 & 0.25 & 0.15 & 0.40 & 0\text{.0}0\\ 0.30 & 0.15 & 0.25 & 0.25 & 0.05 & 0\text{.0}0\\ 0.10 & 0.10 & 0.15 & 0.30 & 0.25 & 0.10\\ 0.15 & 0.35 & 0.40 & 0.05 & 0.05 & 0\text{.0}0\\ 0.10 & 0.20 & 0.30 & 0.30 & 0.10 & 0\text{.0}0 \end{array}\right]R_{5}=\left[\begin{array}{cccccc} 0.15 & 0.10 & 0.35 & 0.15 & 0.25 & 0.00\\ 0.35 & 0.20 & 0.35 & 0.10 & 0\text{.0}0 & 0.00\\ 0.00 & 0.20 & 0.05 & 0.40 & 0.30 & 0.05\\ 0.25 & 0.10 & 0.30 & 0.20 & 0.15 & 0.00\\ 0.00 & 0.10 & 0.10 & 0.35 & 0.45 & 0.00\\ 0.20 & 0.10 & 0.45 & 0.10 & 0.10 & 0.05\\ 0.15 & 0.20 & 0.35 & 0.10 & 0.20 & 0.00 \end{array}\right]\;\;R_{6}=\left[\begin{array}{cccccc} 0.25 & 0.05 & 0.20 & 0.20 & 0.20 & 0.10\\ 0.40 & 0.25 & 0.30 & 0.05 & 0.00 & 0.00\\ 0.30 & 0.25 & 0.00 & 0.20 & 0.25 & 0.00\\ 0.45 & 0.15 & 0.25 & 0.10 & 0.05 & 0.00\\ 0.15 & 0.10 & 0.45 & 0.10 & 0.20 & 0.00\\ 0.20 & 0.20 & 0.20 & 0.25 & 0.15 & 0.00\\ 0.00 & 0.10 & 0.20 & 0.15 & 0.50 & 0.05 \end{array}\right]R_{7}=\left[\begin{array}{cccccc} 0.05 & 0.30 & 0.30 & 0.25 & 0.05 & 0.05\\ 0.25 & 0.35 & 0.30 & 0.10 & 0.00 & 0.00\\ 0.05 & 0.05 & 0.45 & 0.35 & 0.10 & 0.00\\ 0.30 & 0.20 & 0.25 & 0.20 & 0.05 & 0.00\\ 0.05 & 0.00 & 0.30 & 0.30 & 0.35 & 0.00\\ 0.30 & 0.20 & 0.10 & 0.30 & 0.10 & 0.00\\ 0.00 & 0.15 & 0.60 & 0.20 & 0.05 & 0.00 \end{array}\right]\;\;R_{8}=\left[\begin{array}{cccccc} 0.10 & 0.10 & 0.15 & 0.35 & 0.30 & 0\text{.0}0\\ 0.20 & 0.25 & 0.45 & 0.10 & 0.00 & 0\text{.0}0\\ 0.00 & 0.15 & 0.35 & 0.35 & 0.15 & 0\text{.0}0\\ 0.20 & 0.30 & 0.30 & 0.15 & 0.05 & 0\text{.0}0\\ 0.00 & 0.25 & 0.35 & 0.25 & 0.15 & 0\text{.0}0\\ 0.25 & 0.30 & 0.25 & 0.20 & 0.00 & 0\text{.0}0\\ 0.00 & 0.20 & 0.40 & 0.20 & 0.20 & 0\text{.0}0 \end{array}\right]R_{9}=\left[\begin{array}{cccccc} 0.10 & 0.15 & 0.25 & 0.30 & 0.15 & 0.05\\ 0.20 & 0.25 & 0.40 & 0.10 & 0.00 & 0.05\\ 0.00 & 0.05 & 0.45 & 0.45 & 0.05 & 0.00\\ 0.20 & 0.30 & 0.25 & 0.20 & 0.05 & 0.00\\ 0.05 & 0.35 & 0.40 & 0.15 & 0.05 & 0.00\\ 0.30 & 0.30 & 0.35 & 0.05 & 0.00 & 0.00\\ 0.00 & 0.25 & 0.45 & 0.20 & 0.10 & 0.00 \end{array}\right].$$

The evaluation set *P* is:

$$P=\left[\begin{array}{cccccc} 0.0590 & 0.1850 & 0.1850 & 0.2855 & 0.2447 & 0.0408\\ 0.1739 & 0.3478 & 0.2174 & 0.0870 & 0.1304 & 0.0435\\ 0.0815 & 0.1849 & 0.2038 & 0.3261 & 0.1223 & 0.0815\\ 0.1566 & 0.1776 & 0.2350 & 0.1958 & 0.1566 & 0.0783\\ 0.1818 & 0.1604 & 0.2806 & 0.1367 & 0.2004 & 0.0401\\ 0.2077 & 0.1884 & 0.1884 & 0.1662 & 0.1662 & 0.0831\\ 0.1849 & 0.2445 & 0.2445 & 0.2038 & 0.0815 & 0.0408\\ 0.1534 & 0.1740 & 0.1740 & 0.2685 & 0.2301 & 0.0000\\ 0.1700 & 0.1927 & 0.2124 & 0.2549 & 0.1275 & 0.0425 \end{array}\right].$$

According to the principle of maximum membership, the results are shown in Table S3.

### 3.3 SOM neural network clustering analysis

Normalize the data of each indicator. Train the SOM neural network. The number of neurons in the competition layer was set to 5×3 due to the nine milk knot samples. The learning rate of neurons is 0.001, the distance function is linkdist distance, and the number of iterations is set as 10, 20, 50, 100, 200, 500, 1,000 times. In order to facilitate the observation of clustering results, the compet transfer function is used. Save the network and use the network for cluster analysis.

Figure S3 shows the topology of the SOM neural network and the indirect expression of the clustering results (Ma & Liu, 2018). Among them, the regular hexagon represents all competing neurons, while the blue part indicates the winning competition neuron. The number of competition winners is the number of milk knot samples, and the number in the neuron indicates the number of samples possessed by the species.

Figure S4 shows the weight position map of each neuron. Among them, the little dots represent each neuron, and the pentagram represents a sample of milk knot. It can be seen from Figure S4 that the position of the neurons and samples are more dispersed, so they can be classified into a single class according to the difference between each milk knot sample. We can draw a clustering tree based on the connection distance between the winning neurons of the SOM neural network, as shown in Figure S5.

Figure S5 shows the results of cluster analysis of milk knot samples at the connection distance between nine winning neurons in the SOM neural network. Neuron connection distance is a unique classification method in SOM neural network clustering analysis. The two samples with the same degree of similarity will be classified into one class under the smaller neuronal connection distance. Above results showed that samples 1, 3, 8, and 9 are classified into one class, and samples 2, 4, 5, 6, and 7 are classified into another class, which is consistent with the fuzzy hierarchical comprehensive evaluation results. It is accepted that Yili's milk knots are better than Altay's, which indicates that the establishment of a comprehensive evaluation system for the flavor quality of Xinjiang specialty milk knot has certain feasibility. It can successfully evaluate the milk knot of different regions according to the sensible attribute evaluation data to sort.

The creamy aroma, sweet, oxidized aroma, and whey of the Yili's milk knot samples differed greatly from the average of the milk knot samples of Altay's. These four flavor attributes may be the main source of the difference between the two types of milk knot samples. It may be related to the customs, eating habits, and local microbial species of the two regions. However, the difference in sour aroma, milk rancid, and waxy is small between the two categories. The main feature of Xinjiang specialty milk knot is the fermented aroma, the sour taste, the rich waxy, and the faint fragrance, but overall flavor intensity is very high. It has a great relationship with the homemade manual production method of making milk knot, fermented materials (such as sheep intestines), and the taste of Xinjiang local people.

## 4 CONCLUSION

In this study, the homemade milk knots from two different regions of Xinjiang Kazakh ethnic group were used as raw materials to qualitatively and quantitatively descriptive sensory evaluation. The fuzzy AHP based on the optimal consistency matrix was used to comprehensively evaluate the quality of samples, and the quality assessment system of the flavor quality in milk knot samples was explored. Its result is consistent with the cluster analysis of the SOM neural network (Kohara & Enomoto, 2018). The comprehensive evaluation system established by fuzzy analytic hierarchy method is feasible and can be evaluated by flavor quality. The relevant research results can provide a reference for further research on the origin of the difference between the two classes of milk knot samples. The results suggest that this model can be used as a guideline to automated assess the quality of the food and achieve the most important objective of providing a reference for the public and private sectors when making decisions on food quality management (Correa et al., 2018).

## CONFLICTS OF INTEREST

The authors declare no potential conflicts of interest in this study.

## ETHICAL APPORVAL

This article does not contain any studies with human participants or animals performed by any of the authors.

## Supporting information


**Appendix S1**
