# Supplementary material for: Flavor quality evaluation system of Xinjiang milk knots by using SOM neural network and the fuzzy AHP
Source: Food Sci Nutr. 2020 Mar 21;8(4):2088–93. doi: 10.1002/fsn3.1501 (PMC7174216; doi:10.1002/fsn3.1501)
Supplement: Supplementary file 1 — Appendix S1 [file FSN3-8-2088-s001.docx]

Supporting Information for:

Flavor quality evaluation system of Xinjiang milk knots by using SOM neural network and the fuzzy AHP

Zhisheng Wei, Xueping Ma, Ping Zhan^[[1]](#footnote-1)^*, Honglei Tian, Kaixuan Li

**Contents**

1. Figure S1: Xinjiang milk knot sampling location.

2. Figure S2: The result of the sensory evaluation after one-way multivariate analysis of variance.

3. Figure S3: Neural network topology and clustering results.

4. Figure S4: Weight location map.

5. Figure S5: Neuron connection distance clustering tree.

6. Table S1: Xinjiang milk knots sensory attributes.

7. Table S2: Pairwise comparison judgment matrix.

8. Table S3: Evaluation results.


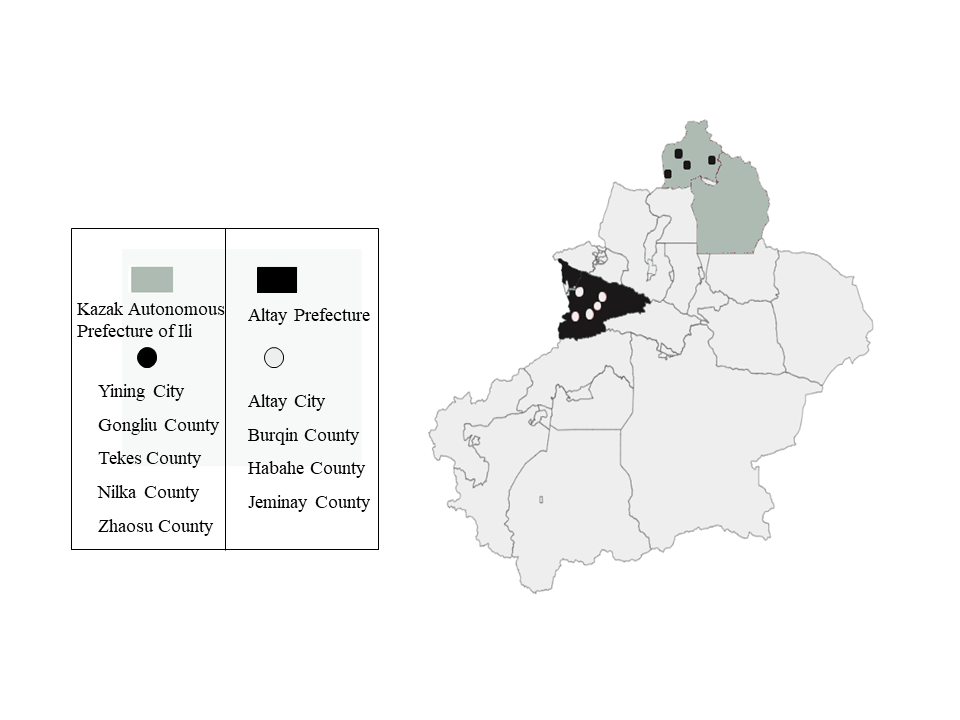


Figure S1: Xinjiang milk knot sampling location


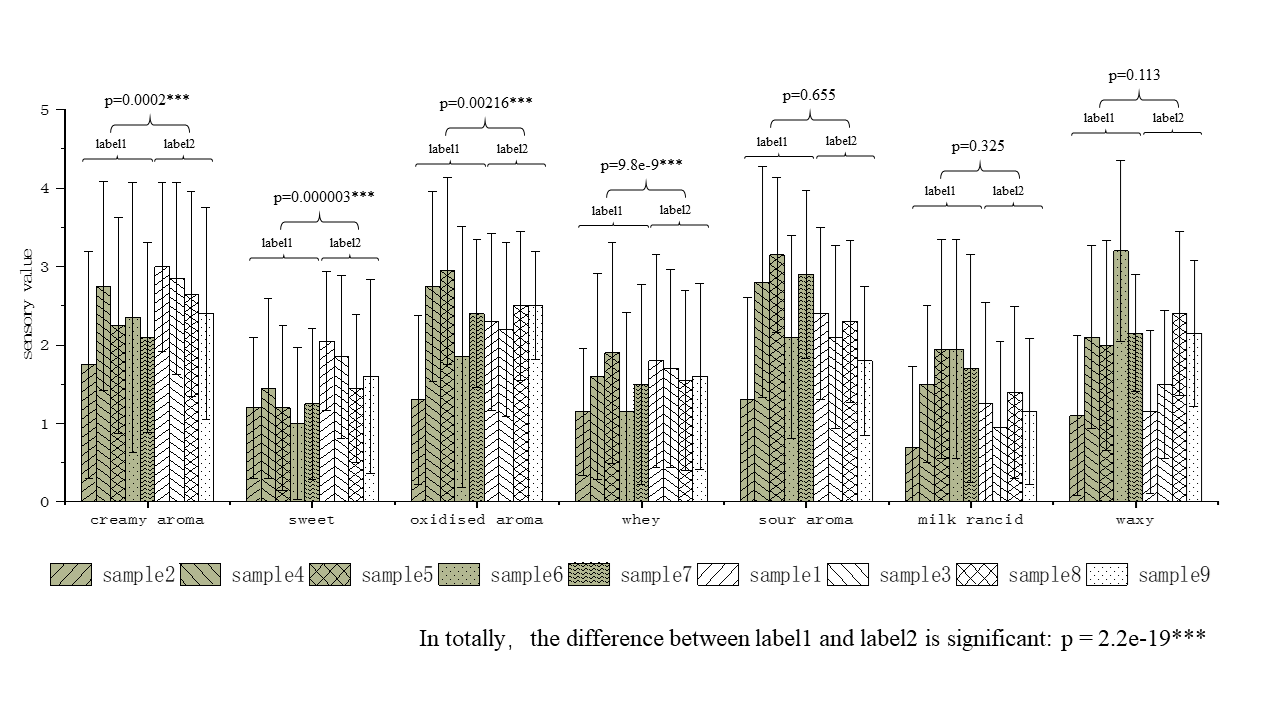


Figure S2: The result of the sensory evaluation after one-way multivariate analysis of variance. The result showed that the categories were significantly different, and the differences in the some sensory qualities were significant. In addition to regional differences, Xinjiang milk knots had something in common, which is the unique flavor of Xinjiang.

**
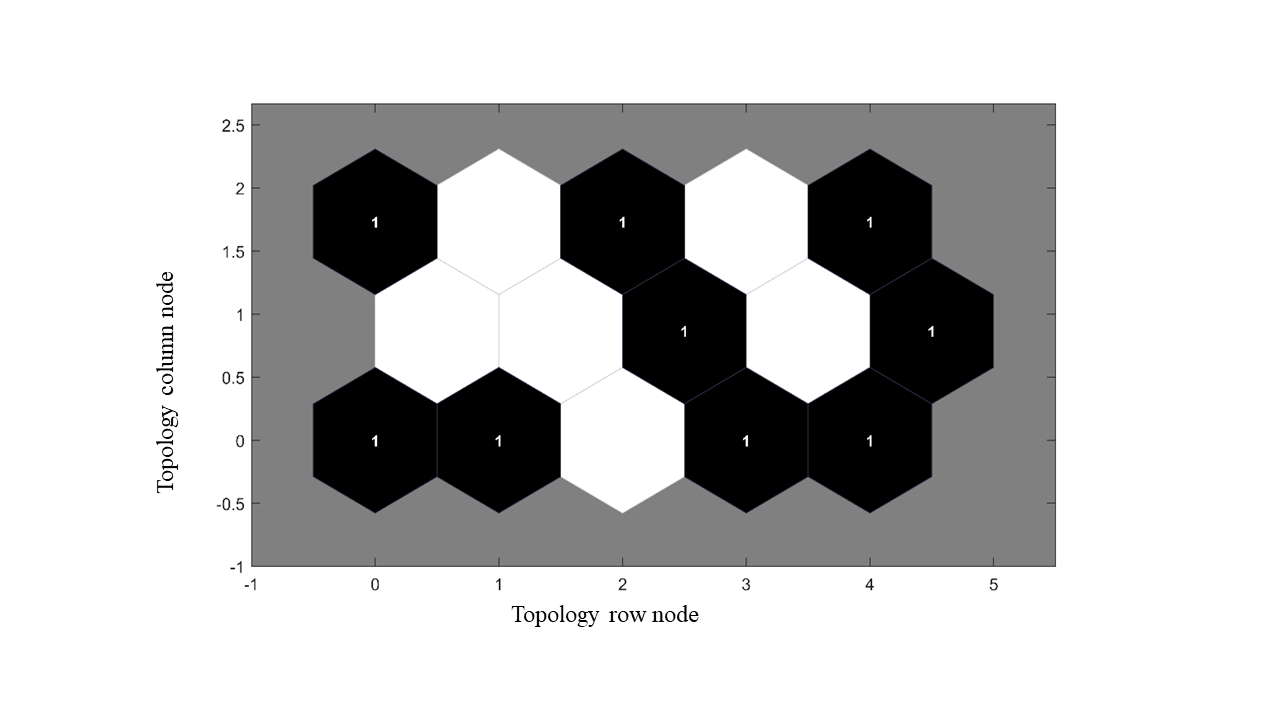
**

Figure S3: Neural network topology and clustering results


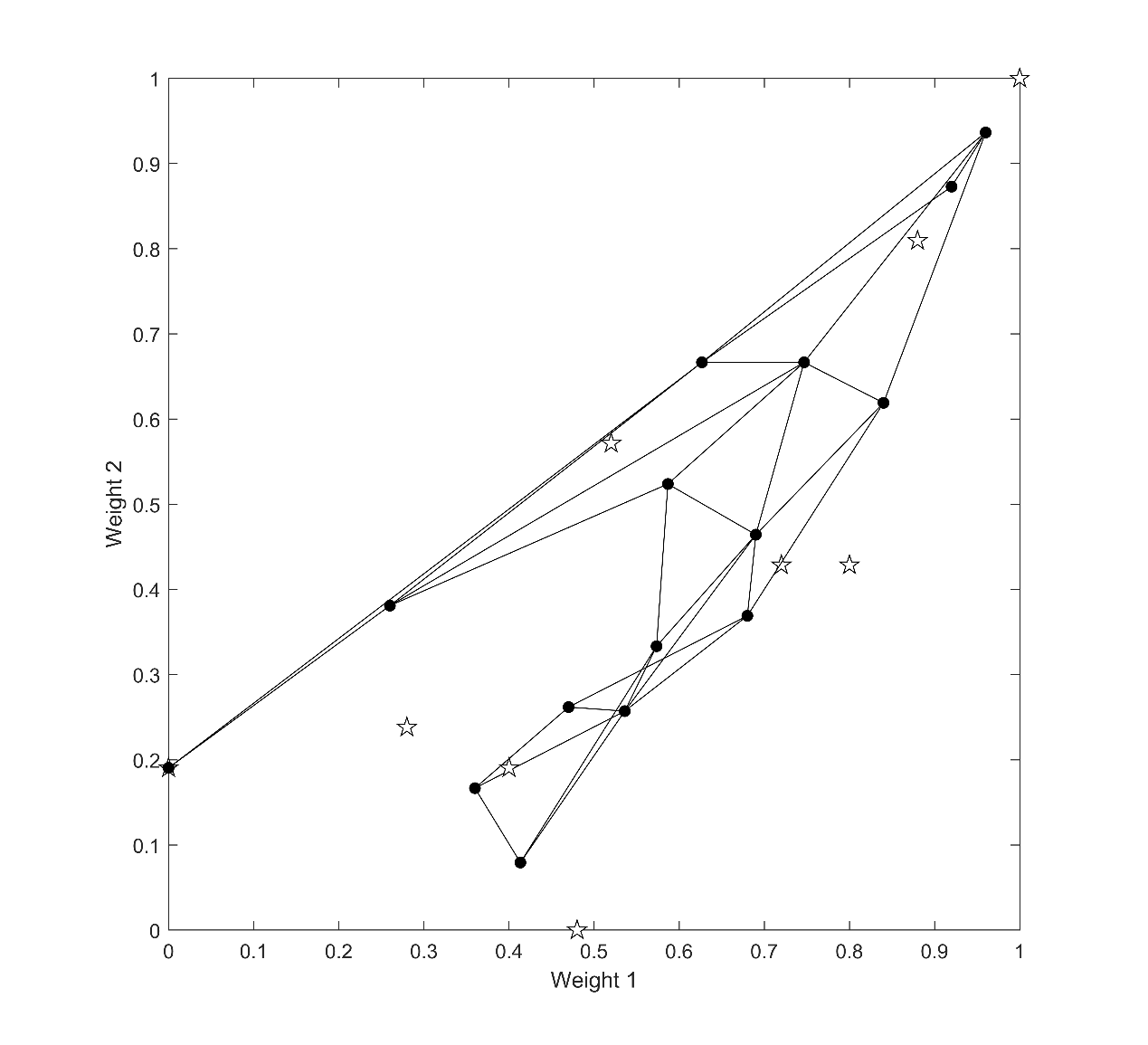


Figure S4: Weight location map


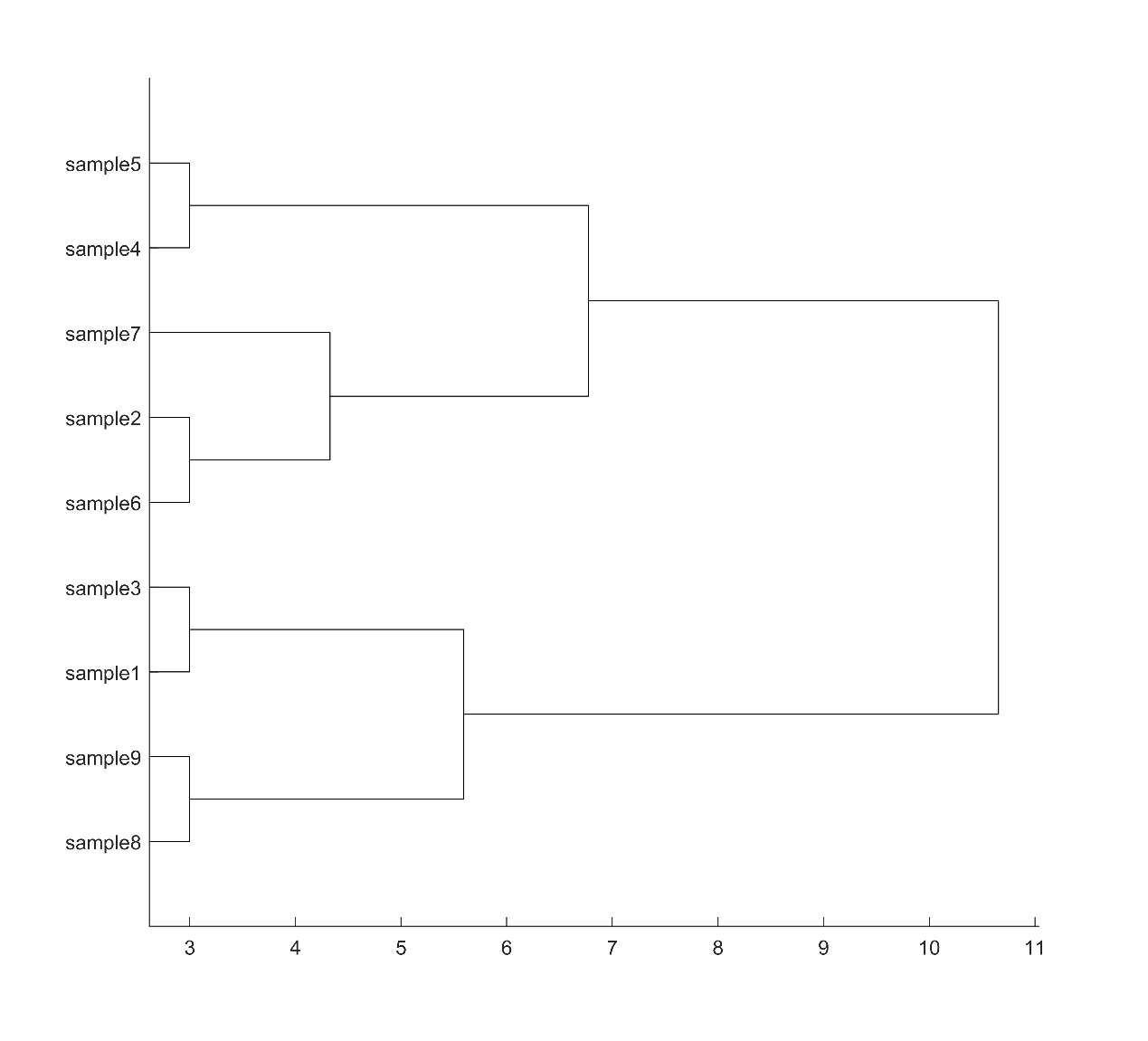


Figure S5: Neuron connection distance clustering tree

Table S1: Xinjiang milk knots sensory attributes

| Attribute | Definition | Scale |
| --- | --- | --- |
| Creamy aroma | The smell associated with creamy/milky products | 5: Unbearable strong smell  4: Strong  3: It is easy to feel the smell  2: The smell is very weak but can distinguish its nature  1: barely feel the smell  0: tasteless |
| Sweet | Aromatics associated with sucrose |  |
| Oxidised aroma | The smell associated with oxidised dairy products |  |
| Whey | Aromatics associated with milk knot whey |  |
| Sour aroma | The aromatics reminiscent of perspiration, foot odor. Sour, stale, moist, stained or odorous with sweat |  |
| Milk rancid | Aromatics associated with boiled meat |  |
| Waxy | Waxy aromatic-primarily associated with milk knots made from sheep's milk |  |

Table S2: Pairwise comparison judgment matrix

|  | creamy aroma | sweet | oxidised aroma | whey | sour aroma | milk rancid | waxy |
| --- | --- | --- | --- | --- | --- | --- | --- |
| Creamy aroma | 0 | 1 | 1 | 2 | 2 | 2 | 2 |
| sweet | -1 | 0 | 1 | 1 | 1 | 2 | 2 |
| oxidised aroma | -1 | -1 | 0 | 1 | 1 | 2 | 2 |
| whey | -2 | -1 | -1 | 0 | 0 | 1 | 1 |
| sour aroma | -2 | -1 | -1 | 0 | 0 | 0 | 1 |
| milk rancid | -2 | -2 | -2 | -1 | 0 | 0 | 0 |
| waxy | -2 | -2 | -2 | -1 | -1 | 0 | 0 |

Table S3: Evaluation results

| samples | Sensory evaluation results of Xinjiang specialty milk knot odor quality |
| --- | --- |
| 1 | 3—good |
| 2 | 1—bad |
| 3 | 3—good |
| 4 | 2—average |
| 5 | 2—average |
| 6 | 0—bad |
| 7 | 2—average |
| 8 | 3—good |
| 9 | 3—good |

1. * Corresponding author:

   Email address: zhanping0993@126.com (P. Zhan) [↑](#footnote-ref-1)
